# TGF-β induces p53/Smads complex formation in the *PAI-1* promoter to activate transcription

**DOI:** 10.1038/srep35483

**Published:** 2016-10-19

**Authors:** Yuki Kawarada, Yasumichi Inoue, Fumihiro Kawasaki, Keishi Fukuura, Koichi Sato, Takahito Tanaka, Yuka Itoh, Hidetoshi Hayashi

**Affiliations:** 1 Department of Cell Signaling, Graduate School of Pharmaceutical Sciences, Nagoya City University, 467-8603 Nagoya, Japan; 2 Department of Innovative Therapeutics Sciences, Cooperative major in Nanopharmaceutical Sciences, Graduate School of Pharmaceutical Sciences, Nagoya City University, 467-8603 Nagoya, Japan

## Abstract

Transforming growth factor β (TGF-β) signaling facilitates tumor development during the advanced stages of tumorigenesis, but induces cell-cycle arrest for tumor suppression during the early stages. However, the mechanism of functional switching of TGF-β is still unknown, and it is unclear whether inhibition of TGF-β signaling results amelioration or exacerbation of cancers. Here we show that the tumor suppressor p53 cooperates with Smad proteins, which are TGF-β signal transducers, to selectively activate *plasminogen activator inhibitor type-1 (PAI-1*) transcription. p53 forms a complex with Smad2/3 in the *PAI-1* promoter to recruit histone acetyltransferase CREB-binding protein (CBP) and enhance histone H3 acetylation, resulting in transcriptional activation of the *PAI-1* gene. Importantly, p53 is required for TGF-β-induced cytostasis and PAI-1 is involved in the cytostatic activity of TGF-β in several cell lines. Our results suggest that p53 enhances TGF-β-induced cytostatic effects by activating *PAI-1* transcription, and the functional switching of TGF-β is partially caused by *p53* mutation or p53 inactivation during cancer progression. It is expected that these findings will contribute to optimization of TGF-β-targeting therapies for cancer.

p53 is the most important tumor suppressor and is inactivated by mutations or deletions in approximately 50% of all malignancies^1^. p53 is activated by various types of stress, and can cause multiple outcomes through different modes of transcriptional activation of its target genes (*e.g.* cell-cycle arrest, DNA repair, and apoptosis)^2^,^3^,^4^,^5^,^6^. For example, p53 induces cell cycle arrest and DNA repair when cells are exposed to low levels of DNA damage, whereas it induces cell death when cells are exposed to extensive DNA damage. Although some p53 effects may be independent of transcription^7^, transcriptional regulation by p53 is important for tumor suppression and loss of its function strongly promotes tumor development^8^.

Transforming growth factor-β (TGF-β) is a multifunctional cytokine that regulates various cellular responses such as cell growth, cell motility, differentiation, apoptosis, and immune-regulation^9^. In cancer, TGF-β acts as tumor suppressor to induce growth arrest, senescence, and apoptosis at the early stages of tumorigenesis, but acts as a tumor promoter to induce epithelial-mesenchymal transition (EMT) and to promote angiogenesis in addition to loss of growth inhibitory effects at the advanced stages of cancer^10^. The tumor-facilitative functions of TGF-β signaling are crucial for high grade of malignancies, and increased TGF-β expression by tumor cells correlates with the progression of colorectal and prostate cancers^11^,^12^. In addition, activation of TGF-β signaling correlates with the resistance to multiple cancer drugs^13^,^14^. Thus, TGF-β signaling switches its functions from tumor suppressive to facilitative during cancer progression^10^. TGF-β signaling is considered to be an attractive molecular target for cancer therapy, and inhibitors of TGF-β signaling, such as receptor kinase inhibitors, neutralizing antibodies, and antisense oligonucleotides, have been used in pre-clinical trials^15^. However, the mechanism of functional switching of TGF-β is still not clear, and identifying this mechanism is important for establishment effective TGF-β-targeted therapeutic strategies for cancer.

TGF-β signaling is transduced into the nucleus by Smad proteins^16^,^17^,^18^,^19^. TGF-β binds a complex of receptors (the TGF-β type I receptor (TβRI) and the TGF-β type II receptor (TβRII)) and activates receptor serine/threonine kinase. Activated TβRI selectively phosphorylates Smad2 and Smad3, resulting in complex formation with Smad4. This complex translocates into the nucleus, where it regulates the transcription of TGF-β target genes through the recruitment of transcriptional coactivators and/or corepressors^20^. Since the affinity of the activated Smad complex to the DNA is insufficient to support association with the promoters of TGF-β target genes, the complex usually requires other DNA-binding factors, so-called Smad cofactors, for eliciting specific transcriptional regulation^21^,^22^,^23^.

Crosstalk between p53 and TGF-β signaling has been reported^24^. Specifically, p53 is required for TGF-β-induced mesoderm differentiation during *Xenopus* embryonic development^25^,^26^ and TGF-β-induced growth arrest in mammalian cells through cooperation with Smads^25^. Cordenonsi *et al.* have shown that several TGF-β target genes were under the joint control of p53 and Smads, and that p53 adjusted TGF-β-induced transactivation by interacting with a cognate binding site on the *Mix.2* promoter^25^. They also found that p53 is required for expression of other TGF-β-induced genes (e.g. *p21*, *PAI-1*, and *MMP2*) through cooperation with Smads, and the presence of a p53 binding site in their promoters^25^. Recently, p53-dependent regulation of *PAI-1* gene expression by TGF-β has been analyzed by the Higgins laboratory^27^. Overstreet *et al.* have shown that TGF-β regulated p53 activity by stimulating p53 phosphorylation and acetylation, promoting interaction with Smads and subsequent binding of the p53/Smads complex to the *PAI-1* promoter^27^. However, the detailed molecular mechanism underlying the crosstalk between p53 and TGF-β signaling has not yet been fully elucidated. Based on these findings, we suggest that p53 acted as a Smad cofactor to enhance the tumor suppressive functions of TGF-β. Here, we focused on the *plasminogen activator inhibitor type-1 (PAI-1*) gene, whose promoter contains both Smad binding element (SBE) and p53 responsive element (p53RE)^27^,^28^,^29^. PAI-1 is required for p53- or TGF-β-induced cellular senescence^30^,^31^. In this study, we revealed that TGF-β induced complex formation between p53 and Smads in the *PAI-1* promoter, and that p53 was required for the recruitment of histone acetyltransferase CREB binding protein (CBP) and the acetylation of histone H3. Moreover, p53 is required for TGF-β-induced cytostatic activity, and PAI-1 is also involved in its effect in several cell lines. These findings suggest that p53 plays an important role in TGF-β-induced cytostatic activity via full activation of *PAI-1* transcription, and that p53 status is involved in the functional switching of TGF-β signaling.

## Results

### p53 enhances TGF-β-induced PAI-1 expression

**Figure 1:**
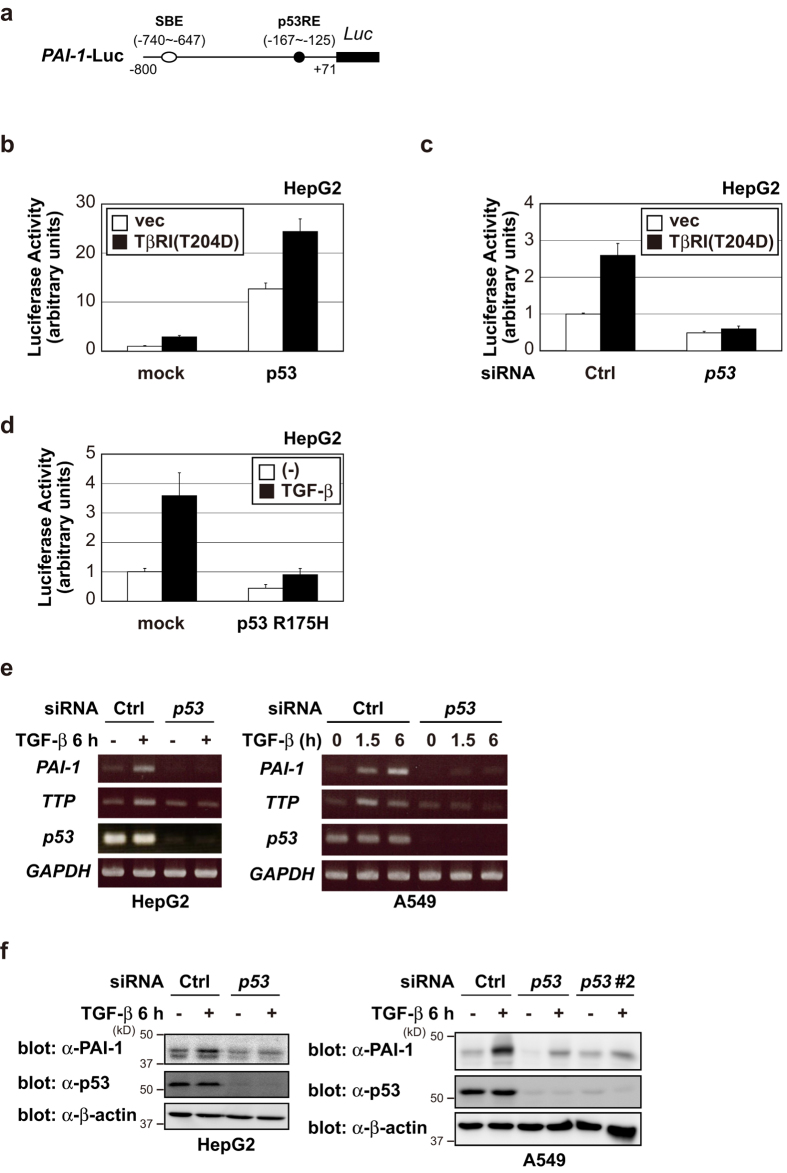
p53 enhances TGF-β-induced PAI-1 expression. (**a**) A schematic representation of the human *PAI-1* promoter construct. (**b,c**) Effects of p53 overexpression (**b**) or p53 knockdown by siRNA (**c**) on the transactivation of *PAI-1* promoter induced by TβRI(T204D) in HepG2 cells. Error bars represent s.d. (**d**) Effects of mutant p53 (R175H) on the transactivation of the *PAI-1* promoter induced by treatment of TGF-β in HepG2 cells. Error bars represent s.d. (**e**) HepG2 cells and A549 cells were transiently transfected with the indicated siRNAs. After 48 h, cells were treated with 100 pM of TGF-β for the indicated periods. Expression of each gene was determined by semi-quantitative PCR. (**f**) HepG2 cells and A549 cells were transiently transfected with the indicated siRNAs. After 48 h, cells were treated with 100 pM of TGF-β for 6 h. The cell lysates were immunoblotted with the indicated antibodies. Uncropped images of gels/blots are shown in Supplementary Information, Figure S1.

Firstly, we performed luciferase assay in HepG2 cells, human hepatoma cell lines expressing wild-type (WT) p53, to investigate the effects of p53 on *PAI-1* transcription. *PAI-1* promoter (−800~ + 71) (Fig. 1a) activity was enhanced by constitutively active TβRI (TβRI(T204D)) expression or p53 expression (Fig. 1b). Interestingly, p53 expression enhanced TGF-β-induced *PAI-1* transcriptional activation. Conversely, transiently knockdown of *p53* by siRNA almost completely suppressed TGF-β-induced *PAI-1* transcription (Fig. 1c), indicating that TGF-β-induced *PAI-1* transcription largely depended on p53. Of note, p53 affected the basal level of *PAI-1* transcriptional activity. This is because p53 itself can activate *PAI-1* transcription. To investigate the effect of p53 mutation on *PAI-1* transcription, we used the mutant p53 R175H. Overexpression of p53 R175H resulted in diminished *PAI-1* transactivation induced by TGF-β (Fig. 1d). Thus, both TGF-β and p53 are necessary for full activation of *PAI-1* transcription in HepG2 cells.

Next, we examined the effects of *p53* knockdown by siRNA on *PAI-1* mRNA and protein expression in HepG2 cells. In addition to the results of the luciferase assay, *p53* knockdown resulted in suppression of TGF-β-induced *PAI-1* mRNA (Fig. 1e, left) and protein expression (Fig. 1f, left). TGF-β stimulation did not significantly affect p53 expression levels. The same result was also obtained using A549 cells, human lung cancer cell lines expressing WT p53 (Fig. 1e,f). Consistent with previous studies^25^,^27^, these results indicate that TGF-β and p53 synergistically regulate PAI-1 expression.

### p53 selectively affects TGF-β target promoters containing both SBE and p53RE

**Figure 2:**
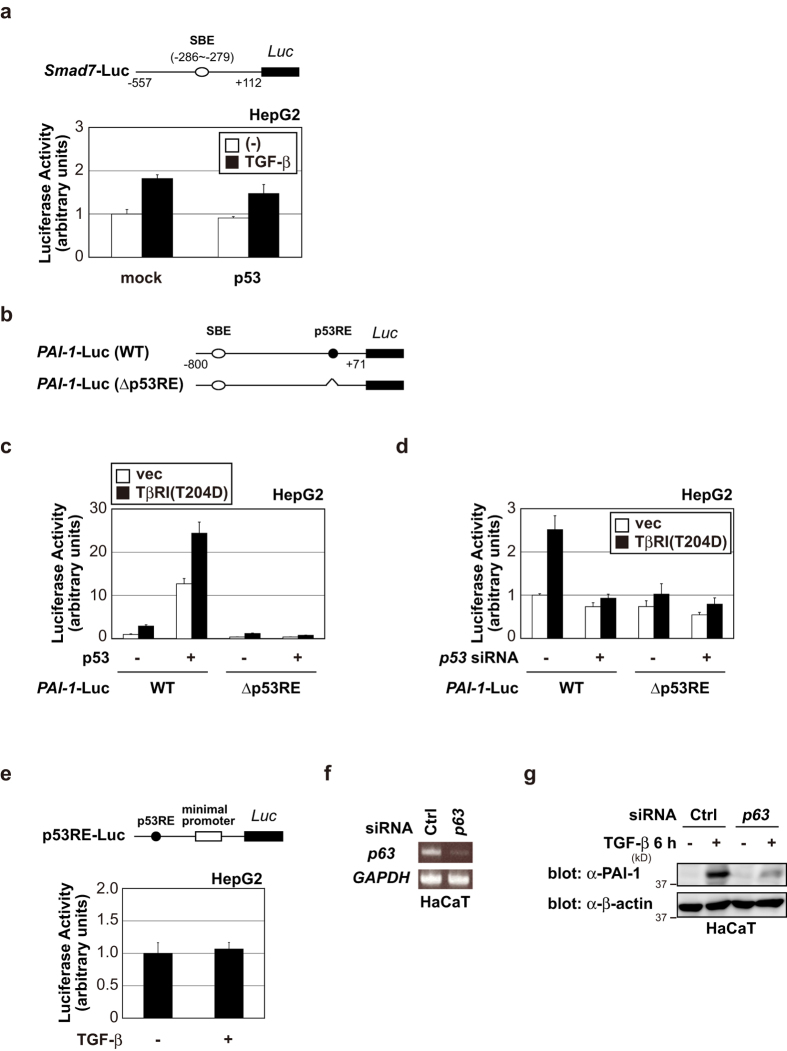
p53 selectively affects TGF-β target promoters containing both SBE and p53RE. (**a**) p53 did not significantly affect TGF-β-induced *Smad7* promoter activation. HepG2 cells were transfected with *Smad7*-Luc in the presence or absence of p53 expression plasmid. After 24 h, cells were treated with 100 pM of TGF-β. After 18 h, luciferase activity was measured. The experiments were performed in triplicate, and the data are represented as the mean-fold activation ± s.d. (**b**) A schematic representation of the human *PAI-1* promoter constructs. (**c**) HepG2 cells were transfected with the indicated constructs. After 24 h, luciferase activity was measured as in (**a**). (**d**) HepG2 cells were transfected with the indicated constructs and siRNAs. After 24 h, luciferase activity was measured as in (**a**). (**e**) TGF-β could not transactivate a p53-responsive reporter. HepG2 cells were transfected with p53RE-Luc. After 24 h, cells were treated with 100 pM of TGF-β for 18 h. The luciferase activity was measured as in (**a**). (**f**) HaCaT cells were transiently transfected with the indicated siRNAs. After 48 h, expression of each gene was determined by semi-quantitative PCR. (**g**) HaCaT cells were transiently transfected with the indicated siRNAs. After 48 h, cells were treated with 100 pM of TGF-β for 6 h. The cell lysates were immunoblotted with the indicated antibodies. Uncropped images of gels/blots are shown in Supplementary Information, Figure S1.

As it has been reported that TGF-β signaling plays multiple roles in tumorigenesis, suppressively or facilitatively, we examined whether p53 affected the transactivation of other TGF-β target genes in HepG2 cells. Interestingly, p53 did not significantly affect TGF-β-induced *Smad7* promoter (−557~ + 112) activation, which is a target promoter region of TGF-β and contains SBE, but not p53RE (Fig. 2a)^32^. This finding suggests that the regulation of TGF-β target genes by p53 depends on the existence of p53RE in these promoters. To demonstrate this more clearly, we constructed a *PAI-1* promoter reporter in which p53RE was deleted (Fig. 2b). In contrast to the WT *PAI-1* promoter, both overexpression and knockdown of p53 hardly affected the mutant *PAI-1* promoter (Δp53RE) activity even after TGF-β stimulation (Fig. 2c,d). Moreover, p53RE-Luc, which contains only p53RE, was unresponsive to TGF-β (Fig. 2e).

Tristetraprolin (TTP) promoter also contains responsive elements for both p53 and Smad^33^,^34^. Therefore, we examined the effects of *p53* knockdown by siRNA on *TTP* mRNA expression in HepG2 and A549 cells. As expected, *p53* knockdown also resulted in suppression of TGF-β-induced *TTP* mRNA (Fig. 1e). Taken together, these results suggested that the synergism of TGF-β signaling and p53 might occur in promoters containing both SBE and p53RE.

p53 inactivating mutations are found in approximately 50% of human cancers^1^. On the other hand, elevated expression of PAI-1 in tumors has been reported^35^. In fact, PAI-1 is up-regulated by TGF-β, even in cell types that carry mutations inactivating p53. This may be explained by other p53 family members, p63 and/or p73, which can compensate for p53 mutation in some cases. Cordenonsi *et al.* have previously shown that *p63* knockdown blunted the induction of p21 by TGF-β in the human keratinocyte cell line, HaCaT (containing mutant H179Y/R828W in p53)^25^. Similarly, *p63* knockdown resulted in suppression of TGF-β-induced *PAI-1* expression in HaCaT cells (Fig. 2f,g). Thus, the regulatory function of p53 to express PAI-1 could be made redundant by the expression of other p53 family members. Alternatively, other Smad cofactor(s) may functionally compensate for mutations causing loss of function of the *p53* gene by cooperating with Smads.

### p53 does not significantly affect TGF-β signal transduction

**Figure 3:**
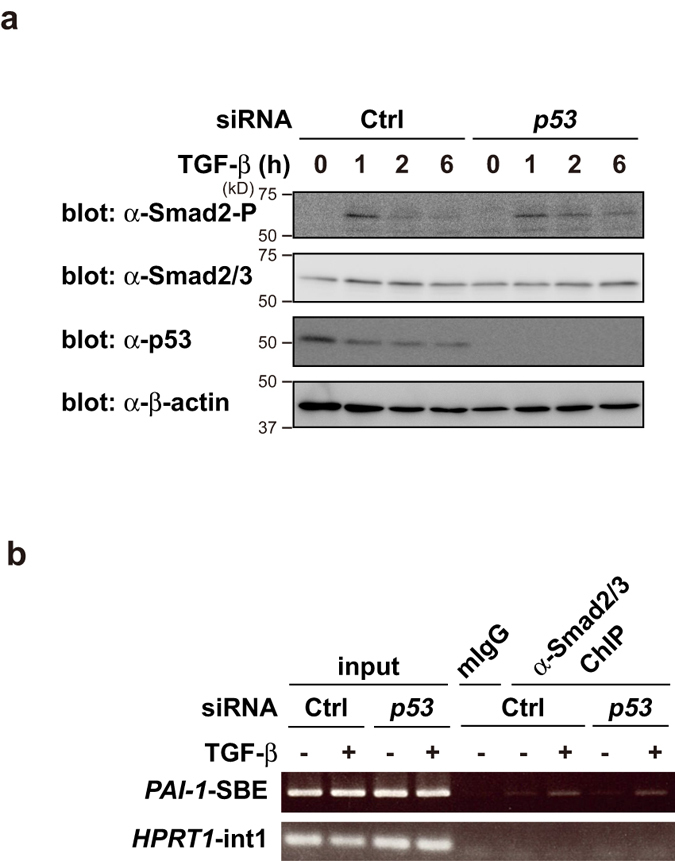
p53 affects neither Smad2 phosphorylation nor DNA-binding activity of Smad2/3. (**a**) HepG2 cells were transiently transfected with the indicated siRNAs. After 48 h, cells were treated with 100 pM of TGF-β for the indicated periods. The cell lysates were immunoblotted with the indicated antibodies. (**b**) HepG2 cells were transiently transfected with the indicated siRNAs. After 48 h, cells were treated with 100 pM of TGF-β for 2 h. The cell lysates were subjected to chromatin immunoprecipitation (ChIP) analysis with the indicated antibodies. Extracted DNA fragments were analyzed by semi-quantitative PCR. Uncropped images of gels/blots are shown in Supplementary Information, Figure S1.

To identify the detailed molecular mechanism of crosstalk between TGF-β signaling and p53 in *PAI-1* transcription, we investigated the effects of *p53* knockdown on Smad2 phosphorylation in HepG2 cells. Smad2 phosphorylation levels were detected by immunoblotting. One hour after TGF-β stimulation, Smad2 phosphorylation reached its peak, and then gradually decreased. TGF-β induced similar levels of Smad2 phosphorylation in both control cells and *p53* knockdown cells (Fig. 3a). We also performed ChIP assay to examine the recruitment of Smad2/3 to the *PAI-1* promoter in HepG2 cells. Consistent with the results in Fig. 3a, *p53* knockdown did not significantly affect Smad2/3 recruitment to the *PAI-1* promoter by TGF-β stimulation (Fig. 3b). Thus, *p53* knockdown does not significantly affect TGF-β signal transduction in HepG2 cells. These findings suggest that p53 might selectively up-regulate *PAI-1* gene transactivation after Smad2/3 binding to the SBE in response to TGF-β.

### The C-terminal domain of p53 interacts with the MH2 domain of Smad3

**Figure 4:**
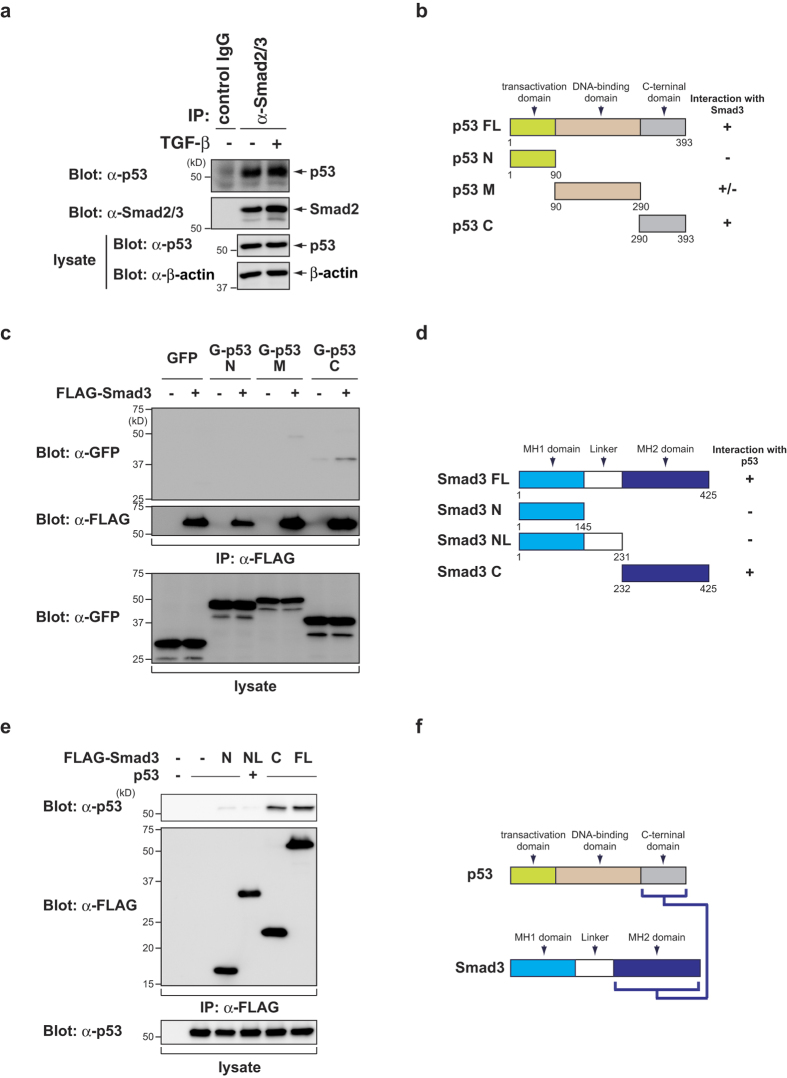
The C-terminal domain of p53 interacts with the MH2 domain of Smad3. (**a**) HepG2 cells were treated with or without 100 pM of TGF-β for 1.5 h. Cell lysates were immunoprecipitated (IP) with an anti-Smad2/3 antibody and then immunoblotted with the indicated antibodies. (**b,d**) A schematic representation of full-length and deletion mutants of p53 (**b**) and Smad3 (**d**). (**c,e**) H1299 cells were transiently transfected with the indicated constructs. After 24 h, the cell lysates were immunoprecipitated (IP) with an anti-FLAG antibody and then immunoblotted with the indicated antibodies. (**f**) A schematic representation of protein-protein interactions between p53 and Smad3. The interacting domains are connected with a blue line. Uncropped images of blots are shown in Supplementary Information, Figure S1.

It has been reported that p53 interacts with Smad2/3 in the presence or absence of the TGF-β family^25^. As shown in Fig. 4a, p53 was co-precipitated with Smad2/3 in HepG2 cells. We next examined p53-Smad3 binding using the deletion mutants of p53 (Fig. 4b). The C-terminal domain of p53 (p53 C), but not other mutants, co-immnoprecipitated with FLAG-Smad3 (Fig. 4c). We also performed immunoprecipitation analysis using various deletion mutants of Smad3 (Fig. 4d). p53 co-immnoprecipitated with the MH2 domain of Smad3 (Smad3 C) (Fig. 4e). Taken together, these findings indicate that the C-terminal domain of p53 interacts with the MH2 domain of Smad3 (Fig. 4f).

### TGF-β induces the complex formation between p53 and Smad3 in the PAI-1 promoter

**Figure 5:**
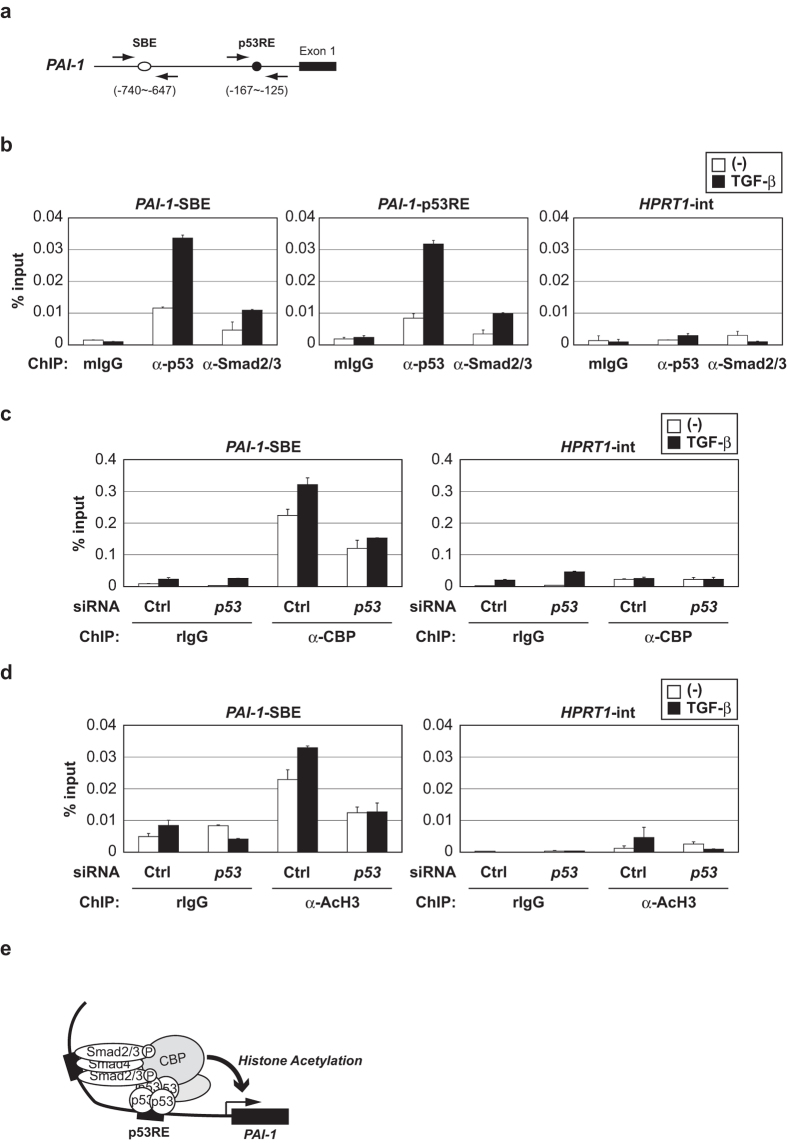
TGF-β promotes p53/Smads complex formation in the *PAI-1* promoter. (**a**) A schematic diagram of a human *PAI-1* gene promoter. (**b**) HepG2 cells were treated with 100 pM of TGF-β for 2 h. The cell lysates were subjected to ChIP analysis with the indicated antibodies. Extracted DNA fragments were analyzed by real-time PCR. Error bars represent s.d. (**c,d**) HepG2 cells were transiently transfected with the indicated siRNAs. After 48 h, cells were treated with 100 pM of TGF-β for 2 h. The cell lysates were subjected to ChIP analysis with the indicated antibodies. Extracted DNA fragments were analyzed by real-time PCR as in (**b**). (**e**) The mechanism of *PAI-1* transcription by the p53/Smads complex. TGF-β induces Smad2/3 to translocate into the nucleus, and the Smad complex interacts with p53 in the *PAI-1* promoter. In other words, p53 plays a role as a DNA-binding partner of Smad. The p53/Smads complex efficiently recruits CBP to the *PAI-1* promoter. The CBP recruitment induces histone H3 acetylation and relaxation of the chromatin structure to activate the *PAI-1* transcription.

We next performed ChIP assay to examine the recruitment of p53 and Smad2/3 in the *PAI-1* promoter (Fig. 5a) by TGF-β stimulation in HepG2 cells. TGF-β stimulation resulted in the recruitment of Smad2/3 to SBE in the *PAI-1* promoter (Fig. 5b, left panel). Interestingly, p53 was also recruited to SBE in response to TGF-β. Similarly, the recruitment of p53 and Smad2/3 to p53RE in the *PAI-1* promoter was induced by TGF-β stimulation (Fig. 5b, middle panel). The recruitment of p53 to SBE suggests that p53 interacts with SBE through Smad2/3, because p53 cannot directly interact with SBE^36^. Similarly it is suggested that Smad2/3 interacts with p53RE through p53. Of note, similar amounts of p53 and Smad2/3 were recruited to both cis-elements by TGF-β stimulation. Consistent with previous studies^27^, it is suggested that TGF-β induces complex formation between p53 and Smad2/3 in the *PAI-1* promoter, and this complex is necessary for TGF-β-induced *PAI-1* transcription. Thus, p53 acts as a partner to Smad for *PAI-1* gene transactivation induced by TGF-β.

### The p53/Smads complex efficiently recruits the transcriptional coactivator CBP to the PAI-1 promoter

It is still unclear why complex formation between p53 and Smad2/3 is necessary for *PAI-1* transcription. We hypothesized that the p53/Smads complex might be required for the recruitment of transcriptional coactivator(s) in the *PAI-1* promoter. We next aimed to examine the coactivator(s) cooperating with the p53/Smads complex.

CBP is a transcriptional coactivator that has histone acetyltransferase activity and cooperates with various transcriptional factors including p53 and Smad^37^,^38^,^39^. Histone acetylation leads to a relaxation of the chromatin structure, and activates transcription. Therefore, we investigated the recruitment of CBP to the *PAI-1* promoter by ChIP analysis in HepG2 cells. TGF-β stimulation resulted in the recruitment of CBP to the *PAI-1* promoter in control siRNA cells. On the other hand, the CBP recruitment in response to TGF-β was diminished in *p53* knockdown cells (Fig. 5c, left panel). In addition, we examined histone H3 acetylation levels using anti-acetylated histone H3 (AcH3) antibody. Similar to CBP recruitment, histone H3 acetylation levels were enhanced by TGF-β stimulation in control siRNA cells, but were suppressed in *p53* knockdown cells (Fig. 5d, left panel). These findings suggest that p53 is necessary for TGF-β-induced CBP recruitment to the *PAI-1* promoter leading to histone H3 acetylation in HepG2 cells. The p53/Smads complex is necessary for the recruitment of CBP and histone acetylation, leading to *PAI-1* transcriptional activation induced by TGF-β (Fig. 5e).

### p53 is required for TGF-β-induced cytostasis in several cell lines

**Figure 6:**
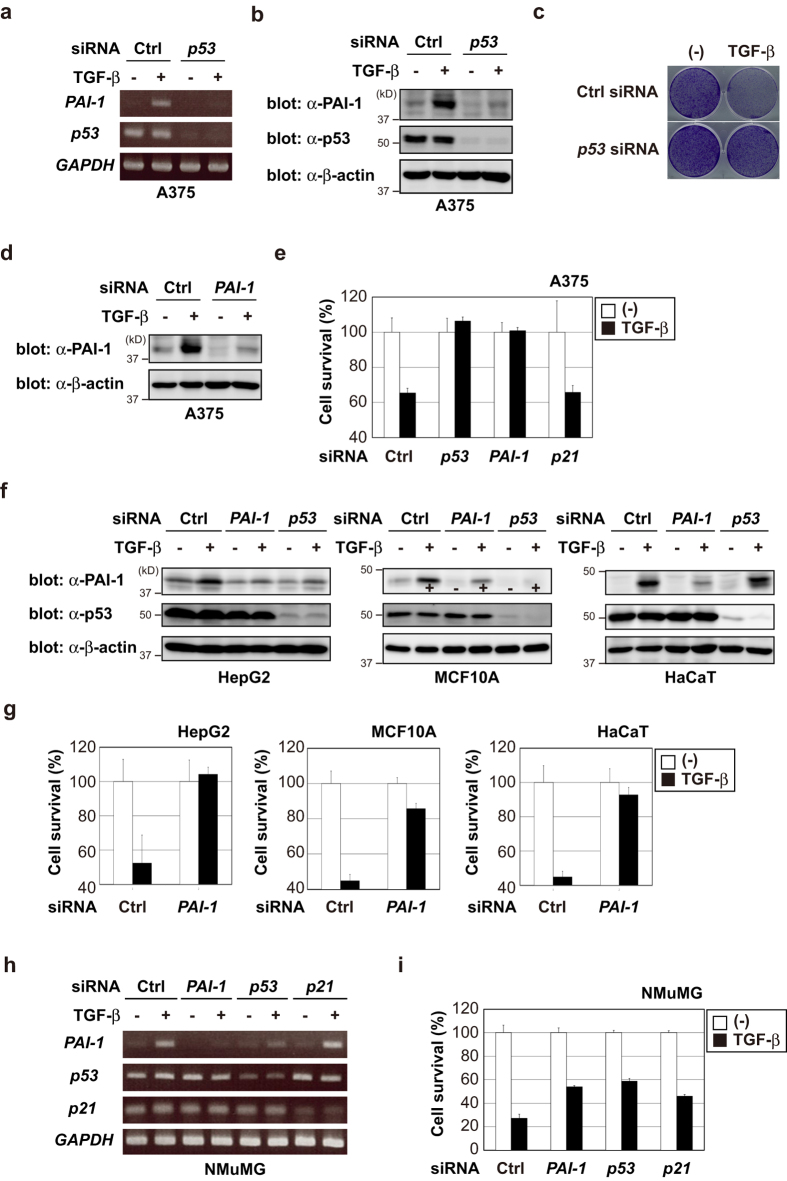
p53 is required for TGF-β-induced cytostasis in several cell lines. (**a,b**) A375 cells were transiently transfected with the indicated siRNAs. After 48 h, cells were treated with 100 pM of TGF-β for 6 h. Expression of each gene was determined by semi-quantitative PCR (**a**). The cell lysates were immunoblotted with the indicated antibodies (**b**). (**c**) A375 cells were transiently transfected with the indicated siRNAs. After 48 h, cells were treated with 100 pM of TGF-β for 96 h and then stained with crystal violet. (**d**) A375 cells were transiently transfected with the indicated siRNAs. After 48 h, cells were treated with 100 pM of TGF-β for 6 h. The cell lysates were immunoblotted with the indicated antibodies. (**e**) A375 cells were transiently transfected with the indicated siRNAs. After 48 h, cells were treated with 100 pM of TGF-β for 72 h and then the cell viability was examined using the CellTiter-Glo assay. (**f**) Cells were transiently transfected with the indicated siRNAs. After 48 h, cells were treated with 100 pM of TGF-β for 6 h. The cell lysates were immunoblotted with the indicated antibodies. (**g,i**) Cells were transiently transfected with the indicated siRNAs. After 48 h, cells were treated with 300 pM of TGF-β for 72 h and then the cell viability was examined using the CellTiter-Glo assay. (**h**) NMuMG cells were transiently transfected with the indicated siRNAs. After 48 h, cells were treated with 100 pM of TGF-β for 6 h. Expression of each gene was determined by semi-quantitative PCR. Uncropped images of gels/blots are shown in Supplementary Information, Figure S1.

It has been shown that TGF-β has a cytostatic effect on human melanoma A375 cells^40^, which express the WT p53. p53 was also required for the induction of PAI-1 by TGF-β, similar to that seen in HepG2 and A549 cells (Fig. 6a,b). Therefore, we investigated whether p53 or PAI-1 was essential for TGF-β-induced cytostasis in A375 cells. As shown in Fig. 6c,e, *p53* knockdown resulted in escape from growth arrest induced by TGF-β. Importantly, *PAI-1* knockdown also resulted in bypass of TGF-β-mediated growth inhibition (Fig. 6d,e). On the other hand, *p21* knockdown did not significantly alter the cytostatic effects of TGF-β.

To strengthen the significance of PAI-1 in TGF-β-mediated cytostasis, the same experiments were performed in other cell lines. HepG2 cells and non-tumorigenic human breast MCF10A cells possess WT p53 and exhibit a strong growth inhibitory response to TGF-β^41^,^42^. As shown in Fig. 6g, the growth of both cell lines was potently inhibited by TGF-β. Similar to A375 cells, *PAI-1* knockdown also decreased the antiproliferative effect of TGF-β (Fig. 6f,g). Consistent with the findings of Kortlever *et al.*^31^, we also found that TGF-β-induced cytostasis was impaired in HaCaT cells (mutant p53 H179Y/R828W) when PAI-1 was knocked down (Fig. 6f,g). Furthermore, we investigated whether PAI-1 is involved in the cytostatic response to TGF-β in a non-transformed mouse mammary epithelial cell line, NMuMG. NMuMG cells biallelically express a WT and a missense mutant (R277C) form of p53^43^. In agreement with a previous report^44^, treatment with TGF-β resulted in decreased proliferation of NMuMG cells (Fig. 6i). *p53* knockdown partially, but significantly, recovered the growth arrest induced by TGF-β (Fig. 6h,i). Likewise, *PAI-1* knockdown in NMuMG cells also partially rescued the decreased proliferation in response to TGF-β (Fig. 6h,i). Of note, *p21* knockdown in NMuMG cells partially reversed the cytostatic effect of TGF-β, in contrast to A375 cells. Collectively, these findings suggest that p53 plays an important role in TGF-β-induced cytostasis via full activation of *PAI-1* transcription, and that loss of p53 function confers resistance to the growth inhibitory activity of TGF-β.

## Discussion

In this study, we reveal the molecular mechanism of TGF-β-induced *PAI-1* transcriptional activation. Specifically, TGF-β induces p53/Smads complex formation in the *PAI-1* promoter, and this complex efficiently recruits CBP to the *PAI-1* promoter, consequently leading to histone acetylation to relax the chromatin structure and activate *PAI-1* transcription (Fig. 5e). Furthermore, these findings suggest that p53 plays a significant role in TGF-β-induced cellular senescence via full activation of PAI-1 expression.

### Not only CBP but also other coactivators possibly enhance TGF-β-induced PAI-1 transcription

Although we clearly show that TGF-β stimulation induced CBP recruitment to the *PAI-1* promoter, it seems that the quantity of CBP recruitment might be insufficient. We hypothesize that p300 or PCAF, which are other transcriptional coactivators of TGF-β signaling^38^,^39^,^45^,^46^, would be recruited to the *PAI-1* promoter in response to TGF-β. Overstreet *et al.* have recently shown transcriptional complex formation involving p53/Smad3/p300 in response to TGF-β^27^. Therefore, it is plausible that TGF-β also causes p53/Smads/p300 complex formation, subsequently resulting in histone acetylation leading to *PAI-1* transcriptional activation. Similarly, it is possible that other transcriptional coactivators and chromatin modifications regulate TGF-β-induced PAI-1 transcription.

### The mechanism of p53 recruitment by TGF-β signaling

Various cellular stresses activate p53-induced transcription by stabilizing and recruiting p53 to target promoters^2^,^3^,^4^,^5^,^6^. In this study, we reveal that TGF-β stimulation induces p53 recruitment to the *PAI-1* promoter without p53 stabilization, but how TGF-β signaling activates p53 is unclear.

We hypothesize that Smad2/3 binding to SBE in the *PAI-1* promoter enhances the affinity between p53 and p53RE. TGF-β signaling could not recruit p53 to the promoter without SBE. TGF-β also induced Smad2/3 recruitment to the *PAI-1* promoter in both control cells and *p53* knockdown cells. Thus, it can be suggested that Smad2/3 binding to the promoter enhances the affinity between p53 and p53RE. In addition, it is also suggested that promoter DNA is necessary for p53/Smads complex formation. Thus, the promoter DNA likely acts as the scaffold for p53 and Smad2/3 to stabilize the p53/Smads complex. This mechanism has been supported by the findings of Cordenonsi *et al.*^25^. p53 altered TGF-β-induced transactivation by interacting with a cognate binding site on the *Mix.2* promoter^25^. Alternatively, it is possible that posttranslational modifications of p53 occur in response to TGF-β. In this regard, it has been reported that TGF-β regulates p53 activity by stimulating p53 phosphorylation and acetylation^27^,^47^.

### p53 selectively enhances TGF-β-mediated tumor suppression

It has been revealed that the promoter of *p21* contains both SBE and p53RE, as well as *PAI-1*^48^,^49^. p21 is a cyclin-dependent kinase inhibitor and induces G1 arrest^49^. p21 is also an effector for TGF-β-mediated tumor suppression. The *TTP* promoter contains responsive elements for both p53 and Smad^33^,^34^, and p53 is required for the induction of TTP by TGF-β. TTP is also known to act as a potent tumor suppressor^50^. We hypothesize that these genes are regulated by the same molecular mechanisms as *PAI-1* transcription. Although further studies are needed to clarify this prediction, we suggest that p53 likely enhances transcriptional activation of various TGF-β target genes related to tumor suppressive functions, such as cellular senescence, cell cycle arrest, and apoptosis.

A previous report has indicated that TGF-β induces EMT in A549 cells, which express WT p53^51^. p53 suppresses the transcription of an EMT-inducing transcriptional factor Snail via the induction of *micro RNA-34a/b/c* genes^52^. Consistent with this, *p53* knockdown enhanced TGF-β-induced Snail expression, but suppressed TGF-β-induced *PAI-1* expression, in A549 cells (data not shown). Therefore, p53 mutation or loss might promote TGF-β-mediated cancer progression and metastasis. Moreover, Huang *et al.* have reported that the activation of TGF-β signaling confers drug resistance in cancer cells via MAPK activation^13^. They examined the effects of TGF-β stimulation on drug resistance in various cancer cell lines. However, the p53 status in those cell lines did not significantly affect TGF-β-induced drug resistance (e.g. WT p53 SKCO-1 cells versus mutant p53 PC-9 cells). Thus, p53 may not be involved in TGF-β-mediated drug resistance, another tumor promoter effect of TGF-β.

Finally, we demonstrated that PAI-1 is involved in the cytostatic response to TGF-β in several cell lines (Fig. 6). Kortlever *et al.* have shown that the induction of PAI-1 by TGF-β is critical for the induction of cellular senescence in HaCaT cells and primary mouse embryonic fibroblasts^31^. They also clarified that PAI-1 is not merely a marker of senescence, but is both necessary and sufficient for the induction of cellular senescence downstream of p53^30^. Mechanistically, PAI-1 expression leads to down-regulation of PI3K-Akt signaling and nuclear exclusion of cyclin D1. Loss of PAI-1 expression or uPA overexpression results in a bypass of cellular senescence^30^. Importantly, although PAI-1 is induced by TGF-β, the growth of most tumor cells is poorly inhibited by TGF-β. One possible explanation is that cells no longer respond to TGF-β-induced cytostatic effects when the PAI-1:uPA (urokinase plasminogen activator) balance shifts toward excess uPA^31^. Overexpression of uPA is often observed in several malignant tumors, and a higher level of uPA expression is associated with poor prognosis^53^. The cellular uPA/PAI-1 ratio would determine whether TGF-β suppresses the tumor growth or not^31^. Another possible explanation is that some cancer driver genes can lead to poor response to PAI-1-mediated cytostatic effects. Further studies are necessary to clarify these possibilities.

In summary, we found p53 to play a crucial role as a Smad partner in TGF-β-mediated tumor suppression, and the functional switching of TGF-β is partially caused by p53 loss or its mutation during tumor development. In future, further investigation into the regulation of TGF-β-mediated tumor-facilitative effects by p53, and identification of the effects of p53 loss or its mutation on TGF-β signaling are needed. In conclusion, we have identified a detailed molecular mechanism in which p53 acts in partnership with Smad to selectively enhance *PAI-1* transcription. Furthermore, p53 might induce selective activation of TGF-β-mediated tumor suppression. Our study helps to clarify the mechanisms of TGF-β functional switching, and ultimately to establish effective TGF-β target therapies for high grade malignancies.

## Methods

### Cell Lines, Plasmids, and Transfections

HepG2, 293, A549, HaCaT, and NMuMG cells were maintained in Dulbecco’s modified Eagle’s medium (DMEM) (Nacalai Tesque) supplemented with 4.5 g/L glucose, 10% fetal bovine serum (FBS) (SIGMA), 100 U/ml of penicillin G, and 100 μg/ml of streptomycin. For culture of NMuMG cells, media were also supplemented with 10 μg/ml of insulin (Wako). H1299 cells were cultured in RPMI1640 medium (Nacalai Tesque) containing 10% FBS and penicillin/streptomycin. MCF10A cells were cultivated in Mammary Epithelial Cell Growth Medium containing bovine pituitary extract, human EGF, human insulin, hydrocortisone (Promocell), penicillin/streptomycin, and choleratoxin (Wako). Cells were grown in a 5% CO_2_ atmosphere at 37 °C.

The original constructs encoding the human p53, Smad3, and TβRI were described previously^54^,^55^. *Smad7*-Luc was kindly provided by Dr. K. Miyazono (University of Tokyo)^32^. pCMV-β-galactosidase (β-gal) was purchased from Clontech. pGL4/p53RE (p53RE-Luc) construct was generated by subcloning a *Asp718*/*HindIII* fragment of pp53-TA-Luc (Clontech) into *Asp718*/*HindIII* of pGL4.10 (Promega). pGL4/PAI-1 (*PAI-1*-Luc) was generated by ligating the human *PAI-1* promoter region (−800~ + 77)^56^ with pGL4.10. pGL4/PAI-1 (∆p53RE) (*PAI-1*-Luc (∆p53RE)) was also constructed by a polymerase chain reaction (PCR)-based approach using pGL4/PAI-1 as a template. All constructs were verified by sequencing.

For DNA transfection, plasmids were transiently transfected with Lipofectamine2000 regent (Invitrogen). For short interfering RNA (siRNA) transfection, siRNAs were transfected using Lipofectamine RNAiMAX reagent (Invitrogen) according to manufacturer’s protocol. siRNA oligo targeting human *p53* has been previously described^54^. The siRNA duplexes were as follows: human *PAI-1* siRNA sense strand, 5′-CCUGGGAAUGACCGACAUGTT-3′^30^; human *p63* siRNA sense strand, 5′-CACACAUGGUAUCCAGAUGTT-3′^57^ mouse *p53* siRNA sense strand, 5′-GUACAUGUGUAAUAGCUCCTT-3′^58^ mouse *PAI-1* siRNA sense strand, 5′-GAACAAGAAUGAGAUCAGUTT-3′^30^ and mouse *p21* siRNA sense strand, 5′-AGACCAGCCUGACAGAUUUTT-3′^59^(SIGMA). siRNA oligo targeting human *p21* (VHS40202) and Stealth RNAi™ siRNA Negative Control Med GC Duplex #2 were obtained from Invitrogen.

### Luciferase Assay

Cells were transfected with the luciferase reporter plasmid, expression plasmids, β-gal expression plasmid, and empty vector. The total amount of transfected DNA was the same in each experiment. Luciferase activity in cell lysates was measured. Luciferase activity was normalized against β-gal activity^60^.

### RNA Extraction and Reverse-transcription

Total RNA extractions were performed as previously described^61^. First-strand cDNA was synthesized with PrimeScript first-strand cDNA Synthesis Kit (TaKaRa Bio Inc.) as previously described^60^.

### Semi-quantitative PCR and Quantitative Real-time PCR

Semi-quantitative PCR was performed as previously described^55^. PCR was performed using AmpliTaq Gold 360 Mater Mix (Applied Biosystems) and a 2720 Thermal Cycler 2700 (Applied Biosystems). The following primer sequences were used: human *p53*, 5′-CTCACCATCATCACACTGGAAGAC-3′ (forward) and 5′-AGAGGAGCTGGTGTTGTTGGGCAG-3′ (reverse); human *GAPDH*, 5′-TGAAGGTCGGAGTCAACGGATTTGGT-3′ (forward) and 5′-CATGTGGGCCATGAGGTCCACCAC-3′ (reverse)^61^; human *PAI-1*, 5′-CATGGGGCCATGGAACAAGG-3′ (forward) and 5′-CTTCCTGAGGTCGACTTCAG-3′ (reverse); human *TTP*, 5′-TCATCCACAACCCTAGCGAA-3′ (forward) and 5′-GATGCGATTGAAGATGGGGA-3′ (reverse)^61^; human *p63*, 5′-CCAGACTCAATTTAGTGAGC-3′ (forward) and 5′-ACTTGCCAGATCATCCATGG-3′ (reverse)^57^; mouse *p53*, 5′-GATGACTGCCATGGAGGAGT-3′ (forward) and 5′-CTCGGGTGGCTCATAAGGTA-3′ (reverse)^62^; mouse *GAPDH*, 5′-GGCATTGTGGAAGGGCTCA-3′ (forward) and 5′-TCCACCACCCTGTTGCTGT-3′ (reverse)^63^; mouse *PAI-1*, 5′-GGGAAAAGGGGCTGTGTGAC-3′ (forward) and 5′-GTACACGGTGTGTGGCTGTC-3′ (reverse)^64^; and mouse *p21*, 5′-TGTCTTGCACTCTGGTGTCTGAGC-3′ (forward) and 5′-TCTTGCAGAAGACCAATCTGCG-3′ (reverse)^65^. PCR amplification was performed in the linear range and PCR products were separated by 1.5–2% agarose gel electrophoresis^55^.

### Antibodies

The following commercially available antibodies used were: anti-PAI-1 (clone 41/PAI-1; BD Biosciences), anti-p53 (DO-1; Calbiochem), anti-β-actin (AC-15; Sigma), anti-phospho-Smad2 (Ser465/467) (138D4; Cell Signaling Technology), anti-Smad2/3 (clone 18/Smad2/3; BD Bioscience), anti-CBP (A-22; Santa Cruz Biotechnology), anti-acetyl-Histone H3 (catalog no. 06–599; EMD Millipore), anti-Myc (4A6; EMD Millipore), anti-FLAG (M2; Sigma), anti-HA (Y-11; Santa Cruz Biotechnology), and anti-GFP (B-2; Santa Cruz Biotechnology). Mouse immunoglobulin G1 (IgG1) (MB002; R & D Systems) and rabbit IgG (Southern Biotech) were used as controls.

### Immunoprecipitation, and Immunoblotting

Immunoprecipitation and immunoblotting were performed as previously described^60^,^66^. The immunoprecipitated FLAG-protein complexes were eluted using 3xFLAG peptide (Sigma) for 30 min on ice.

### Chromatin immunoprecipitation (ChIP) assay

ChIP assay was performed as previously described^54^,^60^. The purified DNA was analyzed by quantitative real-time PCR or semi-quantitative PCR. Quantitative real-time PCR was performed using GeneAce SYBR qPCR Mix α (NIPPON GENE) and a 7300 Real-Time PCR System (Applied Biosystems). For real-time PCR amplification, the following primer sequences were used: human *PAI-1* promoter (SBE), 5′-GCAGGACATCCGGGAGAGA-3′ (forward) and 5′-CCAATAGCCTTGGCCTGAGA-3′ (reverse)^67^; human *PAI-1* promoter (p53RE/TSS), 5′-CCAAGAGCGCTGTCAAGAAGA-3′ and 5′-AGGAATTCAGCTGCTGGAGG-3′ (reverse)^68^; and human *HPRT1* first intron, 5′-TGTTTGGGCTATTTACTAGTTG-3′ (forward) and 5′-ATAAAATGACTTAAGCCCAGAG-3′ (reverse)^54^. For semi-quantitative PCR amplification, human *PAI-1* promoter (SBE), 5′-CCTCCAACCTCAGCCAGACAAG-3′ (forward) and 5′-CCCAGCCCAACAGCCACAG-3′ (reverse)^69^ primers were used.

### Cell Viability assay

Cell viability was measured using the CellTiter-Glo luminescent cell viability assay (Promega) according to manufacturer’s protocol^54^.

## Additional Information

**How to cite this article**: Kawarada, Y. *et al.* TGF-β induces p53/Smads complex formation in the *PAI-1* promoter to activate transcription. *Sci. Rep.* **6**, 35483; doi: 10.1038/srep35483 (2016).

## Supplementary Material

Supplementary Information
