# Supplementary material for: TGF-β induces p53/Smads complex formation in the PAI-1 promoter to activate transcription
Source: Sci Rep. 2016 Oct 19;6:35483. doi: 10.1038/srep35483 (PMC5069723; doi:10.1038/srep35483)
Supplement: Supplementary Information [file srep35483-s1.pdf]

TGF-β induces p53/Smads complex formation in the *PAI-1* promoter to activate transcription

Yuki Kawarada[1,*], Yasumichi Inoue[1,2,*], Fumihiro Kawasaki[1], Keishi Fukuura[1], Koichi Sato[1], Takahito Tanaka[1], Yuka Itoh[1,2] & Hidetoshi Hayashi[1,2]

[1]Department of Cell Signaling, Graduate School of Pharmaceutical Sciences, Nagoya City University; 467-8603 Nagoya, Japan.

[2]Department of Innovative Therapeutics Sciences, Cooperative major in Nanopharmaceutical Sciences, Graduate School of Pharmaceutical Sciences, Nagoya City University; 467-8603 Nagoya, Japan.

*These authors contributed equally to this work.

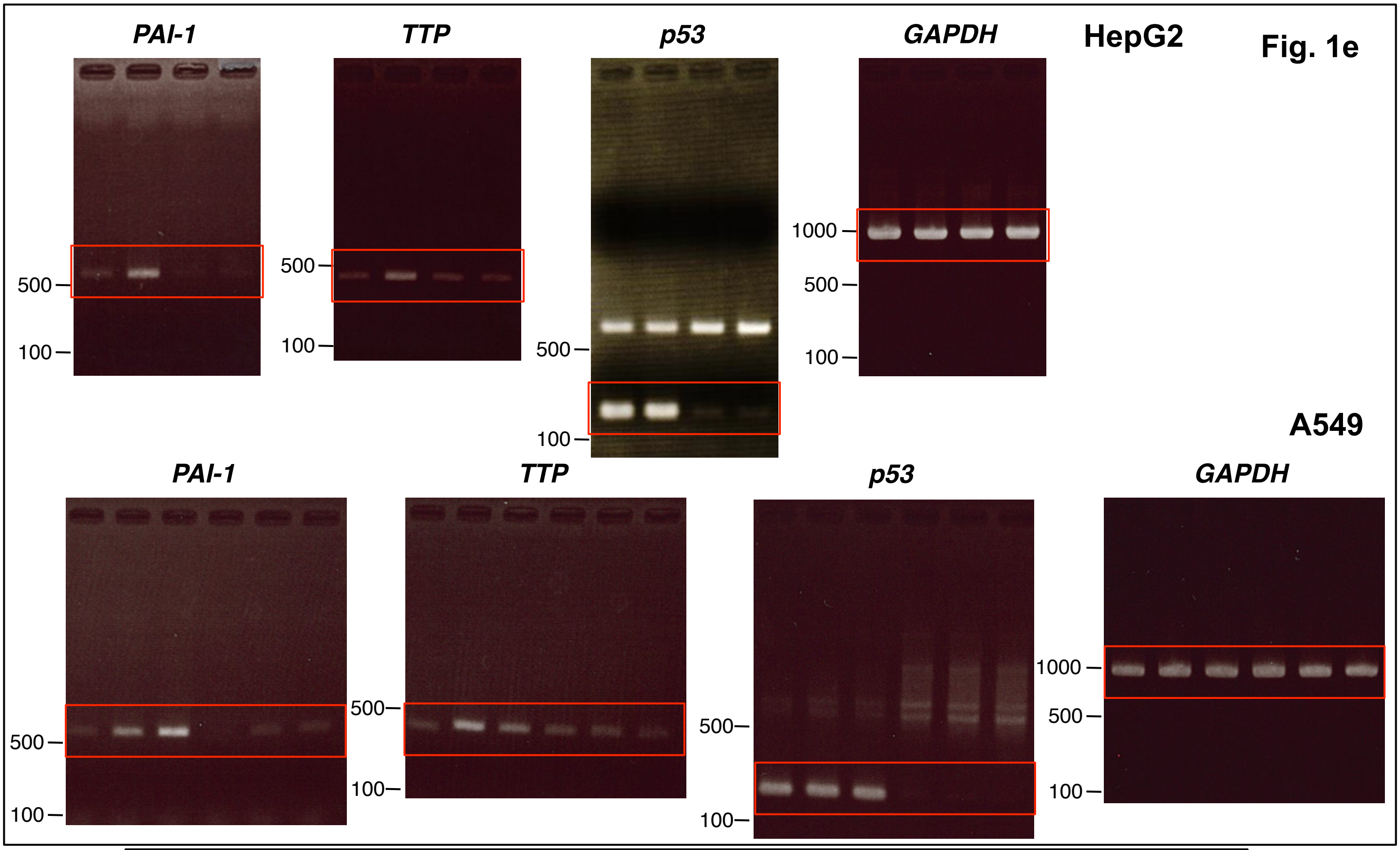

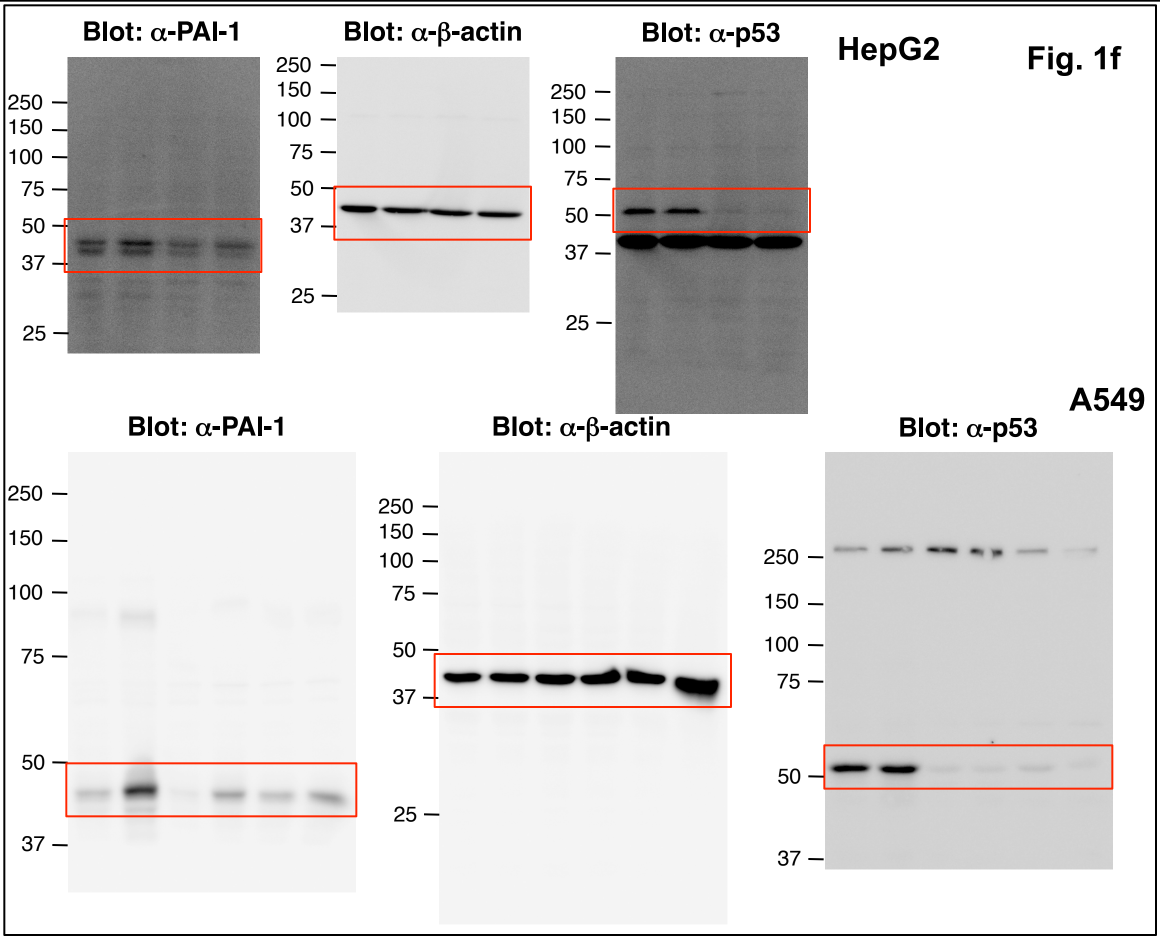

Supplementary Figure 1 Uncropped images of gels/blots
Red boxes indicate the parts that are shown in the figures. Please note that some membranes were cut into multiple strips prior to immunoblotting to detect multiple antigens.

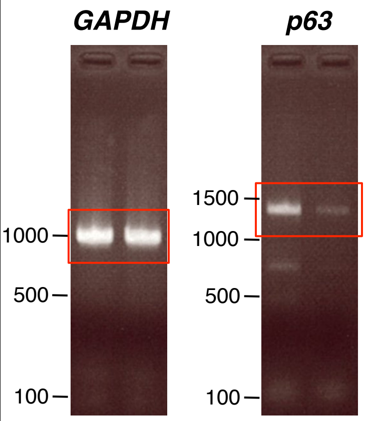

Fig. 2f

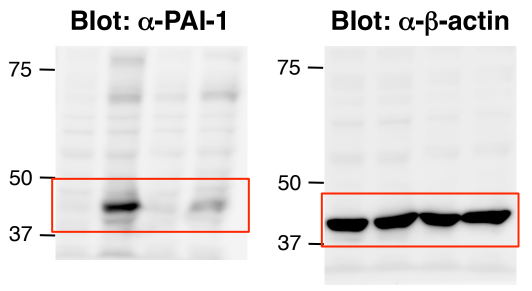

Fig. 2g

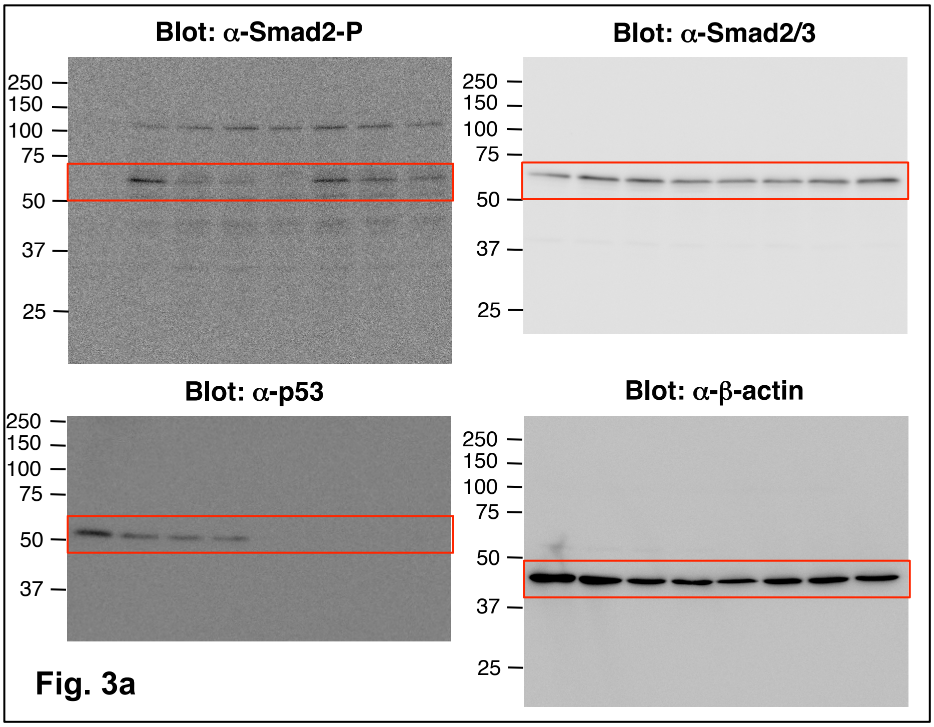

Fig. 3a

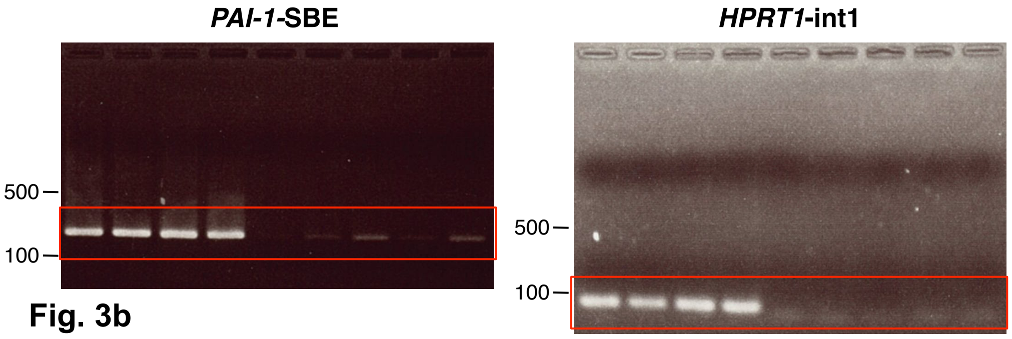

Fig. 3b

Fig. S1 continued

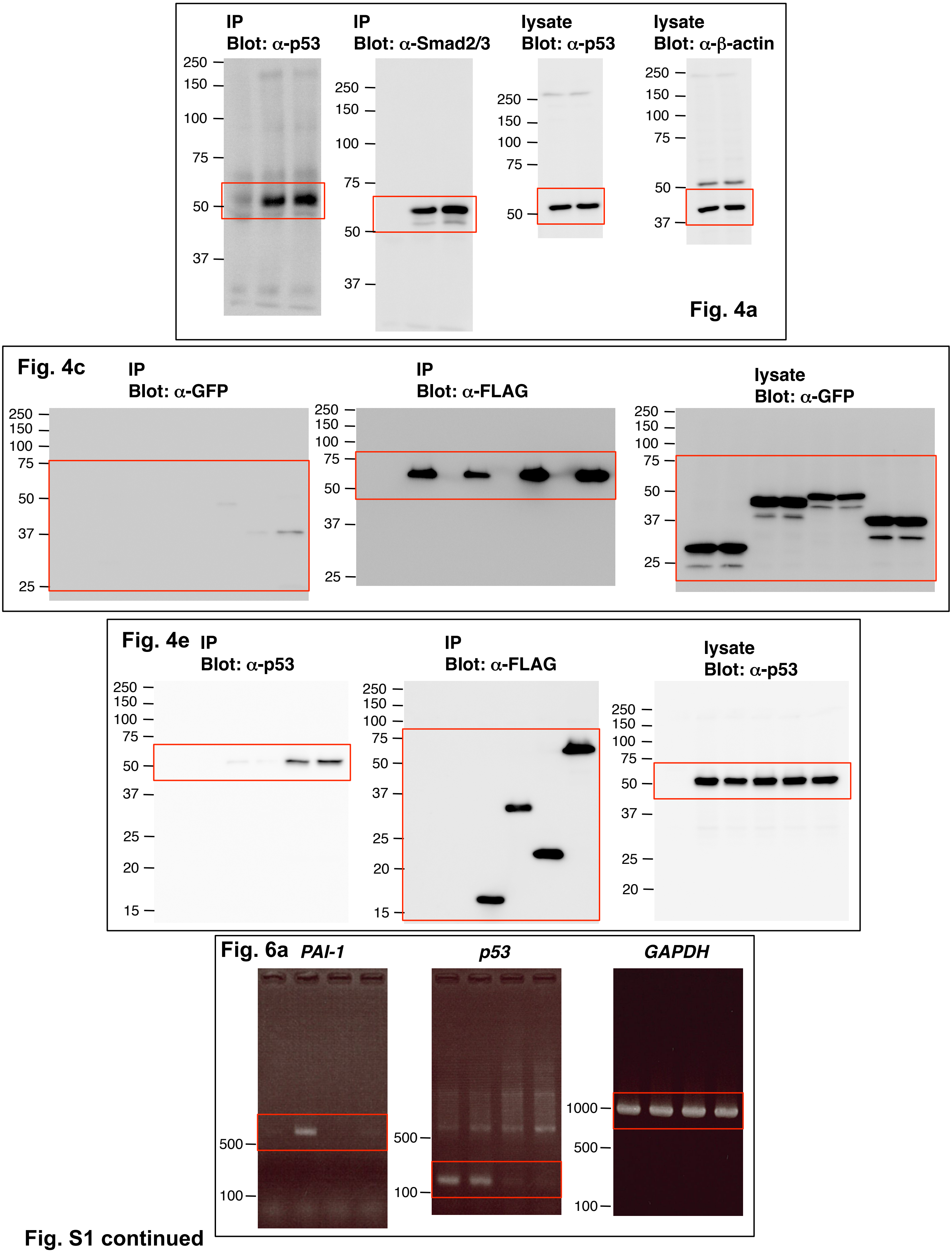

Fig. S1 continued

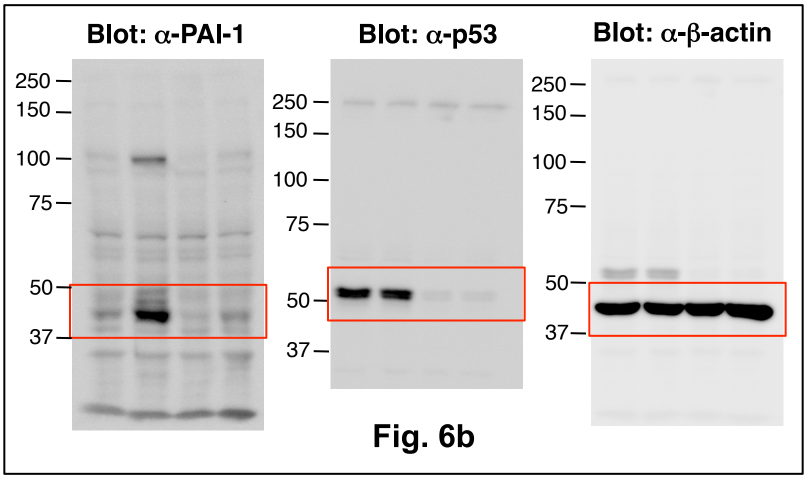

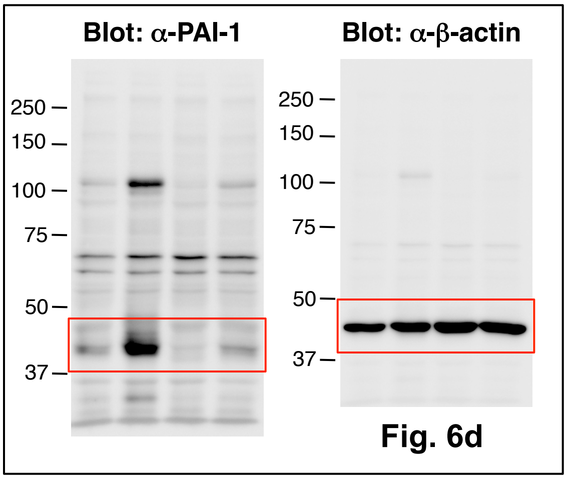

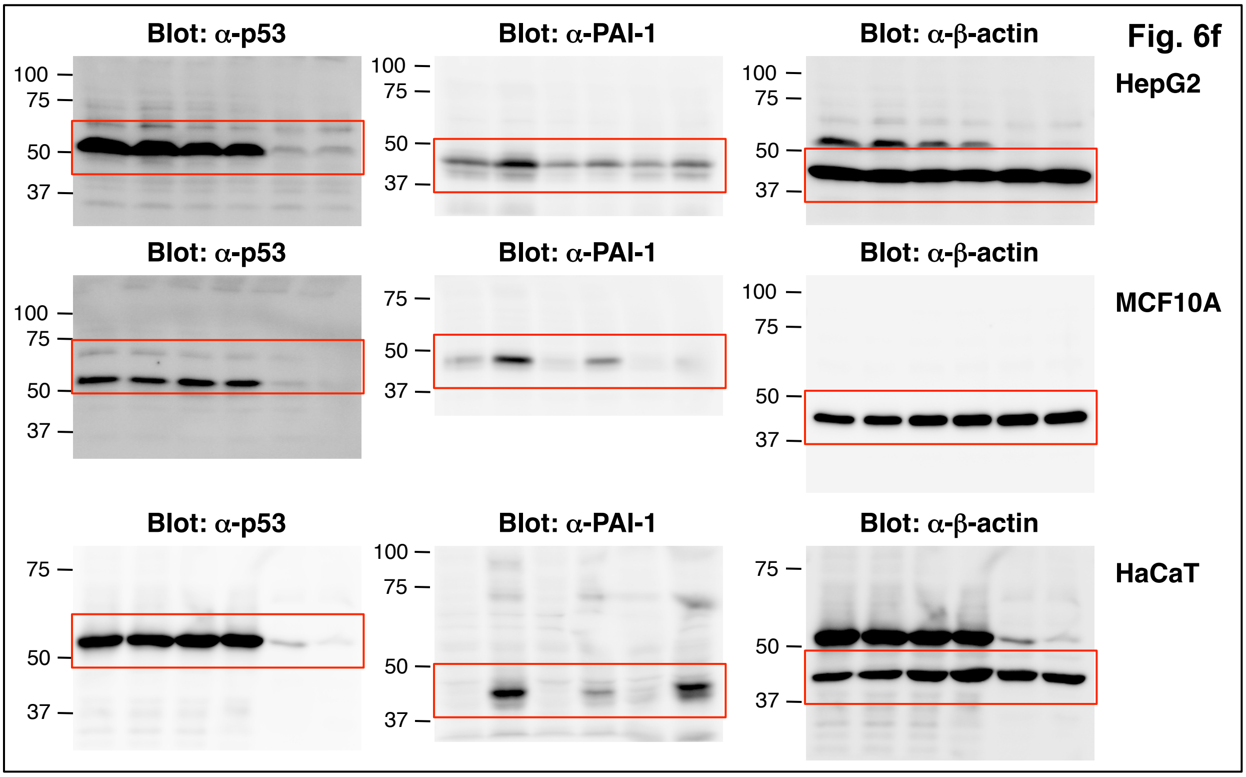

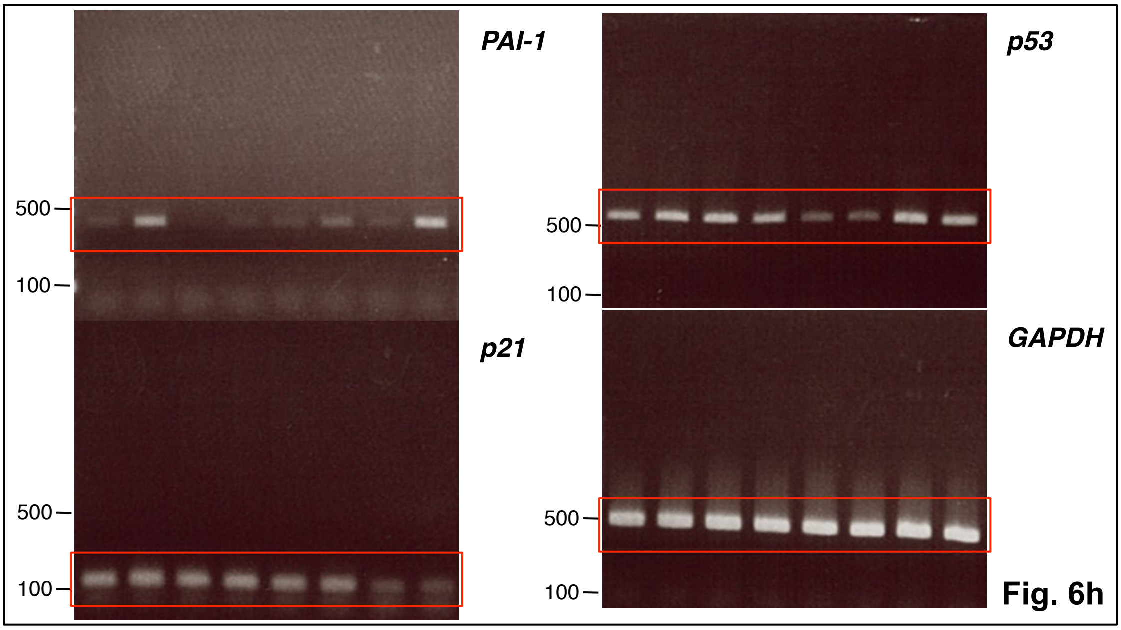

Fig. S1 continued
